# Detecting terahertz wave by microphone based on the photoacoustic effect in graphene foam

**DOI:** 10.1515/nanoph-2023-0026

**Published:** 2023-06-14

**Authors:** Nan Zhang, Tingyuan Wang, Guanghao Li, Lanjun Guo, Weiwei Liu, Ziyuan Wang, Guanghui Li, Yongsheng Chen

**Affiliations:** Tianjin Key Laboratory of Micro-scale Optical Information Science and Technology, Institute of Modern Optics, Nankai University, Tianjin 300350, China; Tianjin Key Laboratory of Metal and Molecule Based Material Chemistry, School of Materials Science and Engineering, National Institute for Advanced Materials, Nankai University, Tianjin 300350, China; Key Laboratory for Functional Polymer Materials and The Centre for Nanoscale Science and Technology, Synergetic Innovation Center of Chemical Science and Engineering (Tianjin), College of Chemistry, Institute of Polymer Chemistry, Nankai University, Tianjin 300071, China

**Keywords:** graphene foam, photo-thermo-acoustic effect, THz detector

## Abstract

Terahertz (THz) wave plays important roles in the research of material properties, the non-invasive human security check and the next generation wireless communication. The progress of the scientific and technological applications of THz wave is strongly dependent on the improvement of THz detectors. Here a novel THz wave detection scheme is proposed in which the THz radiation is detected by an audible microphone based on the photo-thermo-acoustic (PTA) effect in graphene foam. Thanks to the room-temperature broadband electromagnetic absorption characteristics of graphene foam and the fast heat transfer between graphene foam and ambient air, this detection method not only inherits the advantages of the photo-thermal THz detector such as room-temperature and full bandwidth, but also has a response time 3 orders of magnitude faster than the photo-thermal detector. Besides, no micro-antenna/electrode is required to fabricate in the graphene foam THz detector which greatly simplifies the detector design and decreases the fabrication cost. It concludes that the room-temperature, full-bandwidth, fast-speed (≥10 kHz), and easy-to-fabricate THz detector developed in this work has superior comprehensive performances among both the commercial THz detectors and the detectors recently developed in laboratory.

## 1 Introduction

Terahertz (THz) waves are electromagnetic waves with frequencies ranging from 0.1 THz to 10 THz (wavelength 3–0.03 mm). Historically, due to the lack of sources and detectors in this frequency range, there has been little research on THz waves, so this frequency band is also termed of “THz gap”. The special electromagnetic spectrum of THz wave endows it with many excellent characteristics, making THz wave has promising applications in the characterization of atomic/molecular structures [1, 2], the measurement of the ultrafast dynamics of free carriers [3–5], the non-invasive human security check [6–8], the 6G wireless communication [9–14], and remote sensing [15–17].

THz detectors are important devices in the development of THz wave related applications and researches. However, at present, most of the THz detectors are either slow, or operated at low temperature, or has a limited frequency response range [18–20]. Currently it is still challenging for the THz detectors to simultaneously achieve room temperature, broadband, and fast detection. The thermal THz detectors such as bolometer, pyroelectric detector, Golay cell and PTA metal film detector always have a broad frequency detection range at room temperature due to the broadband response of the photo-thermal effect, but suffer from slow detection speed (∼ms). Although bolometers with certain configurations can achieve fast and broadband THz detection [21, 22], low operating temperature is often required. Rectification THz detectors such as Schottky diodes and field effect transistors can achieve extremely fast (∼ns) THz detection at room temperature. However, subjected to the influence of electron transit time and stray capacitance, the response band is typically less than 2 THz. For photon THz detectors such as photoconductive detector, THz wave is absorbed by the electron transition between specific bound states. By measuring electrical parameters such as conductivity/voltage, THz wave can be detected with fast speed (∼ns), however cooling is usually required and the response band is often larger than 1.5 THz [20]. Superconducting kinetic inductance THz detector [23] can achieve extremely high sensitivity and large-scale detection array, however even employing superconductors with high critical temperature [24] it still cannot be operated at room temperature at present. The electro-optic sampling technique based on the pump–probe technique can achieve fast, room-temperature and broadband THz detection, but it requires an ultrafast laser source which is inconvenient for the daily use [2, 4, 25, 26].

The graphene-based THz detector may solve the above dilemma. As a 2D material, the electronic density of states of graphene has sharp peaks near the bottom of the conduction band and the top of the valence band, which benefits the absorption of light with long wavelength, such as THz wave and infrared wave [27, 28]. Moreover, the intraband transition of free carriers in graphene further ensures the strong THz absorption at room temperature under the non-negligible heat excitation [29, 30]. The band structure and intraband transition of graphene guarantee the room-temperature, broadband detection of the graphene-based THz detectors, and the fast photoelectric absorption process makes high speed THz detection become possible. Although in principle utilizing graphene can achieve room temperature, full bandwidth and fast THz detection, at present few graphene-based THz detectors simultaneously possess these advantages [31, 32] due to the limitations in the THz detector scheme and design. For example, the graphene hot electron THz detector can achieve broadband and fast detection, but a low working temperature is always required [33, 34]. In addition, for graphene-based THz detector, the THz energy utilization ratio is low due to the small area and thin thickness of the graphene sheet [34]. The micro-antenna/electrode is always required for graphene detector which further increases the cost of design and fabrication [34, 35].

In this paper, different from the existing THz detection method based on individual graphene sheet, the PTA effect of graphene foam is employed to develop a room-temperature, broadband, fast and easy-to-fabricate THz detector. Graphene foam, i.e. 3D graphene, is a macro-aggregation of many structurally suspended graphene sheets, in which no strong π–π stacking between adjacent graphene sheets exists [36]. Therefore, the graphene foam not only retains the broadband electromagnetic absorption property of graphene, but also has centimeter-scale size in three dimensions, which matches the beam spot of the THz wave and achieves better absorption in THz band. Due to the low heat capacity of graphene foam [37], large temperature difference can be established between graphene foam and ambient air after THz wave is absorbed by graphene foam. The ambient air is heated by graphene foam and acoustic wave is generated if modulated THz wave is applied. The fast heat transfer between graphene foam and ambient air makes the duration of the THz wave-induced acoustic pulse is on the order of microseconds, which guarantees the detection speed of the graphene foam THz detector is three orders of magnitude larger than the existing photo-thermal THz detectors [38, 39]. Besides, not like the THz detector based on the graphene sheet [34, 35], no micro-antennas/electrodes are required in the PTA graphene foam THz detector, which greatly simplifies the detector structure, decreases the fabrication cost, and could expedite practical and commercial applications. Utilizing the PTA effect in graphene foam, the THz wave can be directly measured by a commercial microphone with audible response bandwidth, achieving the response time of 8.4 μs and the noise equivalent power (NEP) of 182 nW/Hz^0.5^. The performances of the PTA THz detector proposed in this paper are much better than the existing commercial PTA THz detector which has millisecond-scale response time and NEP of 5 μW/Hz^0.5^ [40]. Furthermore, the responsivity and NEP of the PTA THz detector investigated in this paper may be further improved when more sensitive acoustic wave detection method, such as the optical micro-ring resonator [41] or fiber optic acoustic sensor [42], are employed.

## 2 Results

### 2.1 Concept design

**Figure 1:**
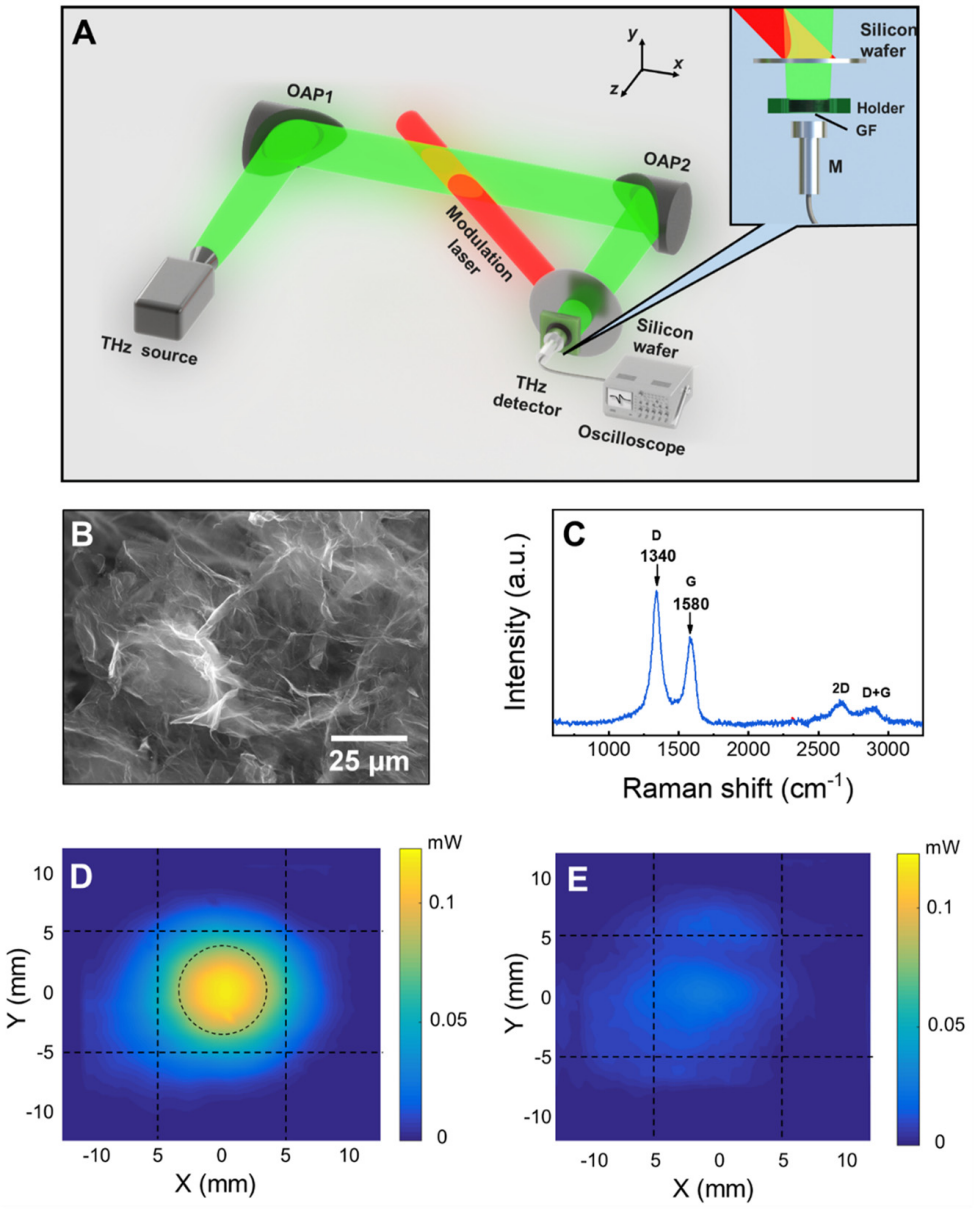
Illustration of the graphene foam THz detection scheme. (A) Configuration of the PTA graphene foam THz detector (see the inset) and the setup used to demonstrate its validity (top view); OAP, off-axis parabolic mirror; GF, graphene foam; M, microphone; (B) and (C) are the SEM picture and the Raman spectrum of the graphene foam, respectively; (D) and (E) are the cross-sectional intensity profile of the THz wave on the front surface of the graphene foam after passing through the unmodulated and modulated silicon wafer, respectively. The power of the modulation laser is 30 mW.

The configuration of the PTA THz detector and the apparatus used to demonstrate its validity are shown in Figure 1(A). A 0.1 THz, 86 mW THz source (Terasense Inc.) is employed to emit the continuous THz wave via a conical horn. The THz wave is collimated by an off-axis parabolic mirror (OAP1, its open aperture’s diameter and reflected focal length are 3 inches and 6 inches, respectively) and then focused by another identical off-axis parabolic mirror (OAP2) onto the surface of the graphene foam. The cylindrical graphene foam sample used in experiments has a diameter of 7.0 mm and a thickness of 2.0 mm which is held by the sample holder. The scanning electron microscopic (SEM) image and the Raman spectrum of the graphene foam are respectively shown in Figure 1(B) and (C). It is seen that the graphene foam is composed of many suspended graphene sheets and retains most of the properties of graphene. More details about the graphene foam can be found in the “Materials and methods” section.

In order to achieve the PTA energy conversion in graphene foam, the temperature of the graphene foam must be changed periodically under the irradiation of the amplitude modulated THz wave. Through the periodic heat transfer between graphene foam and ambient air, air undergoes repeated expansion and compression, producing acoustic waves. Therefore, like other THz thermal detectors, the PTA graphene foam THz detector responds only to the modulated THz wave.

The amplitude modulated/pulsed THz wave can be generated by modulating the THz source using an arbitrary waveform generator (DG3101A, Rigol Inc.). The experimental results are presented in Figure S1 of the Supplementary Material [43]. It is found that the responsivity of the PTA graphene foam THz detector is independent on the modulation frequency (up to 10 kHz) of THz wave. However, this modulation method cannot change the peak-to-peak amplitude of the modulated THz wave, which is critical for studying the responsivity and noise equivalent power (NEP) of the detector. Therefore, in the main text of this paper the laser excited silicon wafer is used to modulate THz wave. In Figure 1(A), a 0.5-mm-thick intrinsic silicon wafer irradiated by the modulation laser (35 fs, 50 Hz, 800 nm collimated Gaussian laser pulse train) with an incident angle of 45° is placed between OAP2 and the graphene foam to modulate the amplitude of the THz wave. When the silicon wafer is irradiated by the modulation laser, the free carrier density increases, leading to the decrease of the transmittance of THz wave. Since the modulation laser consists of a pulse train with a repetition rate of 50 Hz, both the free carrier density and the THz transmittance of the silicon wafer change periodically. Therefore, the amplitude of the THz wave after passing through the silicon wafer is modulated at a repetition rate of 50 Hz.

The cross-sectional intensity profile of the THz wave on the silicon wafer is measured by a zero-biased Schottky diode detector (WR10ZBD, Virginia Diodes Inc.). The experimental apparatus and the measurement result are shown in Figure S2 of the Supplementary Material [43]. It shows that the cross-sectional intensity distribution of the THz wave on the silicon wafer is nearly Gaussian with beam diameters (1/*e* ^2^) of 14.8 mm and 13.6 mm respectively along *x* and *y* axis. The intensity distribution of the modulation laser on the silicon wafer is also measured which is shown in Figure S3 of the Supplementary Material [43]. The laser beam diameters (1/*e* ^2^) are, respectively, 11.8 mm and 8.3 mm along *x* and *y* axis. The beam spots of both the THz wave and the modulation laser on the silicon wafer are larger than the diameter of the graphene foam.

**Figure 2:**
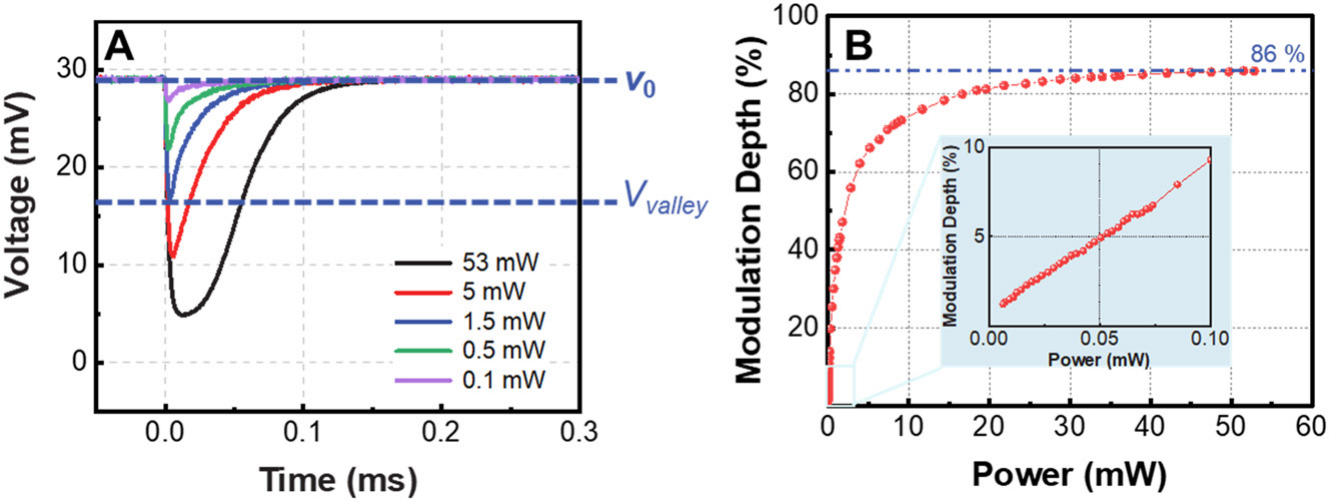
Performance of the modulated THz waves. (A) Time-dependent THz power passing through the laser excited silicon wafer when the modulation laser with different power is used; the voltage in the vertical axis is proportional to the THz power which is measured by the Schottky diode detector; (B) dependence of the modulation depth of the THz power on the power of modulation laser. The inset shows the zoom-in plot for low power modulation laser.

The intensity profile of the THz wave on the front surface of graphene foam after passing through the unexcited silicon wafer is measured and shown in Figure 1(D). The dashed circle in Figure 1(D) shows the location of the graphene foam. Therefore, by integrating the THz intensity inside the dashed circle, the total THz power incident on the graphene foam is calculated to be 10.4 mW. Figure 1(E) presents the measured cross-sectional intensity profile of the THz wave on the graphene foam when the silicon wafer is irradiated by the modulation laser with a power of 30 mW. Since the Schottky diode detector has a fast detection speed of 250 kHz, for each point in Figure 1(E), a time-dependent curve showing the temporal variation of the THz power passing through the laser excited silicon wafer just like those shown in Figure 2(A) is measured. The lowest THz power in the curve is used as the transmitted THz power plotted in Figure 1(E). In this case, the total THz power on the graphene foam is calculated to be 1.7 mW. Therefore a 30 mW modulation laser can induce 8.7 mW THz power variation. From Figure 1(D) and (E), it also concludes that the entire THz wave striking on the graphene foam is modulated by the laser excited silicon wafer.

The modulated THz wave strikes on the graphene foam and the audible acoustic wave is measured by a microphone (378C01, PCB Inc.). The acoustic frequency range that can be detected by the microphone is from 4 Hz to 100 kHz. The temporal profile of the acoustic wave is recorded by an oscilloscope (DPO3034, Tektronix Inc.).

### 2.2 Performance characterization of the PTA THz detector

During the experiments, the power of the modulation laser ranges from 0.01 to 53 mW. The time-dependent THz power transmitting through the laser-excited silicon wafer is measured by the Schottky diode detector (WR10ZBD, Virginia Diodes Inc.). A conical horn antenna (WR10CH, Virginia Diodes Inc.) is added in front of the Schottky diode detector so that all the transmitted THz power can be collected by the detector (see Figure S4 of the Supplementary Material [43]). The Schottky diode detector used here has a detection speed of 250 kHz and a frequency response band from 0.075 THz to 0.11 THz. The measurement results are shown in Figure 2(A). The voltage in the vertical axis of Figure 2(A) is proportional to the THz power. Therefore, the power modulation depth (*MD*) of the THz wave can be calculated by *MD* = (*V*_0_ − *V*_valley_)/*V*_0_, where *V*_valley_ is the voltage at the valley of the curve in Figure 2(A) and *V*_0_ is the voltage corresponding to the case that the THz wave is not modulated. The calculated dependence of the modulation depth of the THz power on the modulation laser’s power is shown in Figure 2(B). It is found that as the laser power increases, the THz wave’s modulation depth tends to saturate and the upper limit of MD is 86 %. It should be noted that since the Schottky diode detector has a detection speed of 250 kHz, i.e. a response time of 4 μs, the time-dependent curves in Figure 2(A) are the temporal profile of the modulated THz waves.

**Figure 3:**
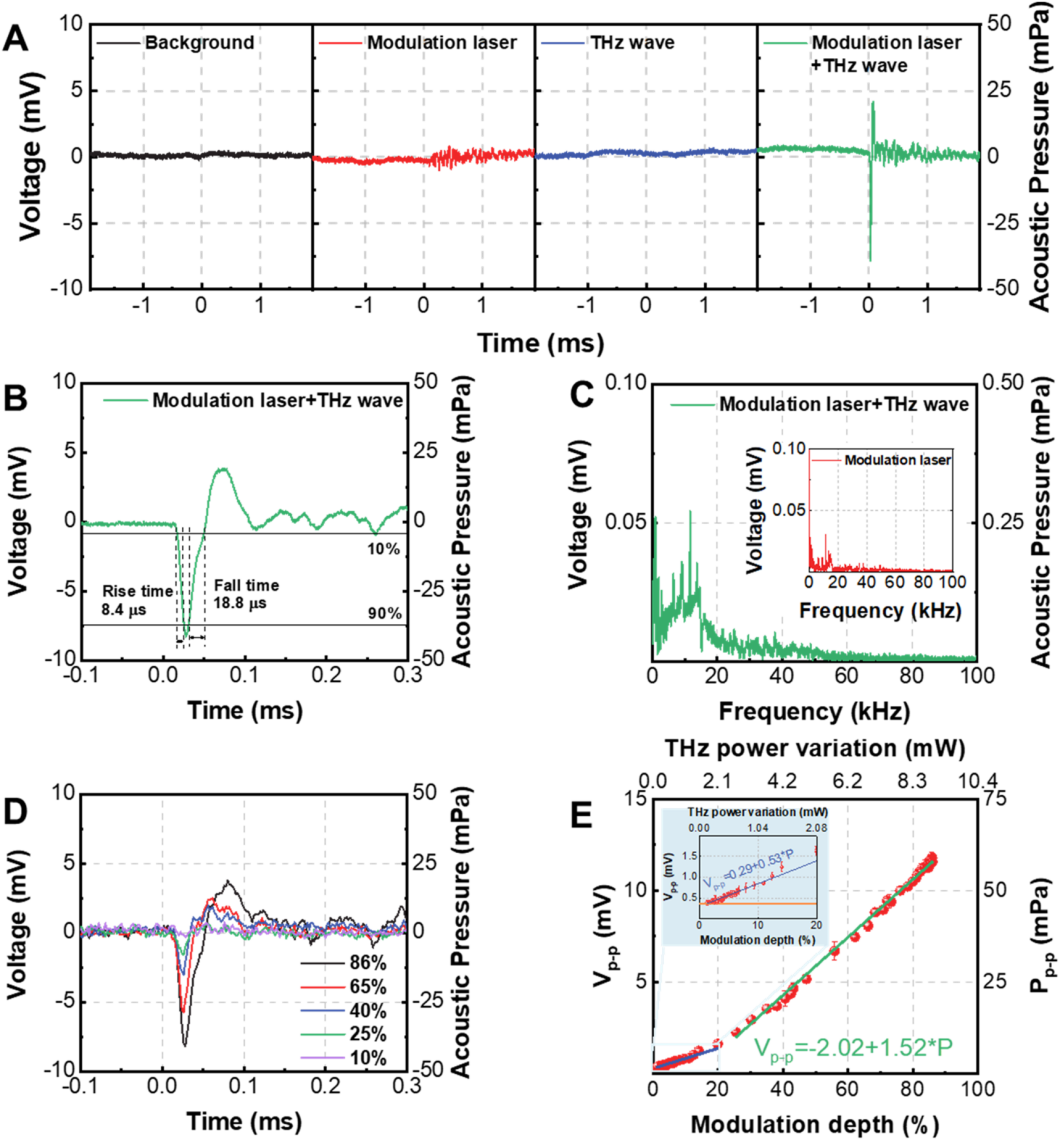
Performance of the PTA THz detector. (A) Acoustic signal measured by the microphone under four different experimental conditions; (B) zoom-in picture of the acoustic pulse in (A) when the THz wave and modulation laser are simultaneously employed; (C) frequency spectrum of the acoustic pulse in (B), the inset is the frequency spectrum of the acoustic signal when only the modulation laser is employed, i.e. the one shown in the second picture from the left in (A); (D) acoustic pulses generated by the modulated THz waves with different modulation depths; (E) dependence of the peak-to-peak pressure of the acoustic pulse on the modulation depth or power variation of the THz wave. The inset shows the zoom-in plot for *MD* ≤ 20 %. The orange horizontal line in the inset indicates the background noise induced voltage when blocking the THz wave.

Using the setup in Figure 1(A), the background noise is firstly recorded by the microphone when both the modulation laser and the THz wave are blocked. The measured background noise is presented as the black curve in Figure 3(A). Secondly, when only the modulation laser with a power of 30 mW is employed to irradiate the silicon wafer, acoustic noises can be detected by the microphone which is shown as the red curve in Figure 3(A). Thirdly, when the modulation laser is blocked and only the continuous THz wave irradiates the graphene foam, only the background noise is measured and shown as the blue curve in Figure 3(A). Contrastingly, when the 30 mW modulation laser and the THz wave are simultaneously employed, the acoustic pulse emitted from the graphene foam is detected and presented by the green curve in Figure 3(A). The voltage measured by the microphone used in the experiments is proportional to the acoustic pressure and the proportionality coefficient is constant over 4 Hz–100 kHz. According to the results in Figure 2(B), the 30 mW modulation laser can generate a THz modulation depth of ∼84 % and thus the acoustic pulse indicated by the green curve in Figure 3(A) is induced by a THz power variation of 8.7 mW.

The acoustic pulse (green curve in Figure 3(A)) is zoomed in and shown in Figure 3(B). It is seen that the rising time and falling time of the acoustic pulse are respectively 8.4 μs and 18.8 μs. The acoustic pulse is generated due to the sudden decrease and increase of THz wave’s amplitude (see Figure 2(A)) which is caused by the excitation and relaxation of the free carriers in the silicon wafer irradiated by the modulation laser. By comparing Figures 2(A) and 3(B), it is found that the duration of the acoustic pulse is nearly identical to the duration of THz power variation. By Fourier transforming the acoustic pulse’s temporal profile in Figure 3(B), the frequency spectrum of the acoustic pulse is presented in Figure 3(C), showing that the most part of the acoustic energy locates below the audible upper limit of 20 kHz. The inset in Figure 3(C) is the frequency spectrum of the acoustic signal when only the modulation laser is employed (see red curve in Figure 3(A)). By comparing these two spectra, it can deduce that the sharp spikes around 10 kHz are acoustic noises generated by the interaction between the modulation laser and the silicon wafer.

The acoustic pulses generated by the modulated THz waves with different modulation depths are presented in Figure 3(D) and the dependence of the peak-to-peak pressure of the acoustic pulse on the THz power variation is summarized in Figure 3(E). The THz power variation is calculated by $\Delta P_{\text{THz}}\left(MD\right)=P_{\text{THz}}\times MD$ , where Δ*P*_THz_(*MD*) indicates the THz power variation when the modulation depth equals to *MD*, and *P*_THz_ is the THz power incident on the graphene foam when no modulation laser is employed. *P*_THz_ = 10.4 mW in our experiments. Figure 3(E) infers that the peak-to-peak pressure of the acoustic pulse can be used as the evaluation of the THz power’s variation. By linearly fitting the data, it is found that the responsivities of the detector are 0.53 V/W and 1.52 V/W, respectively, for the THz power variation less or larger than 2.1 mW. The slight increase of air temperature in the vicinity of graphene foam may be the reason why the detector has smaller responsivity for THz power variation less than 2.1 mW. The smaller the modulation depth is, the more THz power is deposited in the graphene foam, and the less thermal energy is dissipated by the acoustic wave, which may lead to the increase of air temperature. According to the theoretical model of the thermoacoustic effect [44], the acoustic pressure *P*_acoustic_ is proportional to (*α*_air_)^1/2^ *ρ*_air_/*T*_air_, in which *α*_air_, *ρ*_air,_ and *T*_air_ are, respectively, the thermal diffusivity, density, and temperature of air. It is found that as *T*_air_ increases, (*α*_air_)^1/2^ *ρ*_air_/*T*_air_ decreases, leading the decrease of the acoustic pressure and the responsivity.

NEP with unit of Watt is defined as the input THz power with a signal-to-noise ratio (SNR) of 1. The orange and blue lines in the inset of Figure 3(E) are, respectively, the voltage of background noises and the voltage generated by the input THz power. Therefore, the intersection of the two lines means SNR = 1. Since NEP is proportional to the square root of the measurement bandwidth, for the convenience of comparing between different detectors, NEP is always normalized to 1 Hz bandwidth. Therefore, by dividing the input THz power (i.e. THz power variation) at the intersection of orange and blue lines in the inset of Figure 3(E) by the square root of the 100 kHz measurement bandwidth of the microphone, average NEP with unit of W/Hz^0.5^ is obtained. The NEP is determined to be 345 nW/Hz^0.5^ in this case.

Since the duration of the acoustic pulse detected by the microphone is on the order of tens of microseconds, the THz detector in Figure 1(A) can achieve ≥10 kHz fast detection and the experimental results are shown in Figure S1 of the Supplementary Material [43]. By comparing Figures 2(A) and 3(D), it is found that the duration of the acoustic pulse roughly equals to the duration of the THz power variation.

The fast detection speed of the graphene foam PTA THz detector is mainly contributed by the fast heat transfer between the graphene foam and the ambient air. The heat flow between the solid film and ambient gas can be calculated by *Q* = *hS*D*T* ∝ *hS*/*C*_*ua*_ [45, 46], where *h* and Δ*T* are, respectively, the convective heat-transfer coefficient and temperature difference between the solid film and ambient gas, *S* and *C*_*ua*_ are, respectively, the surface area and heat capacity per unit area (HCPUA) of the solid film. Most of the building blocks of the graphene foam used in our experiments are single-layer or two-layer graphene [47], which has an extremely low *C*_*ua*_ [44, 48] of 5.8 × 10^−4^ J m^−2^ K^−1^ or 1.16 × 10^−3^ J m^−2^ K^−1^. Therefore, the THz energy deposition in graphene foam can lead to a large temperature difference between the graphene foam and ambient air. Meanwhile, the graphene foam used in experiments has a density of ∼1 mg/cm^3^ and a specific surface area of 2.4 × 10^2^ m^2^/g [49], leading to a very large contact area *S* ∼ 180 cm^2^ with ambient air. Contrastingly, for the conventional PTA THz detector using metallic film as the PTA conversion material, to guarantee over 50 % THz absorptance [40], at least 10-nm-thick chromium or 90-nm-thick aluminum film is needed [50, 51], whose *C*_*ua*_ is 3.2 × 10^−2^ J m^−2^ K^−1^ or 2.2 × 10^−1^ J m^−2^ K^−1^, respectively, much larger than that of graphene foam. Besides, the contact area between the metallic film and ambient air is in the order of ∼10 cm^2^, 1 order of magnitude smaller than that of graphene foam. Therefore, although the convective heat-transfer coefficient *h* between graphene with ambient air is 12.4 J s m^−2^ K^−1^ [45], slightly smaller than that of metallic film [52] (for example *h* between chromium and ambient gas is 17.1 J s m^−2^ K^−1^ [45]), the low *C*_*ua*_ induced large temperature difference *DT* and large surface area *S* of graphene foam result in the fast heating of adjacent ambient air layer. Based on the relation of *Q* = *h*D*TS* ∝ *hS*/*C*_*ua*_, the heat flow between graphene foam and ambient air is 2–3 orders of magnitude larger than that of metallic film. The fast heat transfer results in the fast generation of acoustic pulses with duration 2–3 orders of magnitude shorter than that of metallic film PTA THz detector, leading to a much faster detection speed.

### 2.3 Enhancement of the responsivity of the PTA THz detector

**Figure 4:**
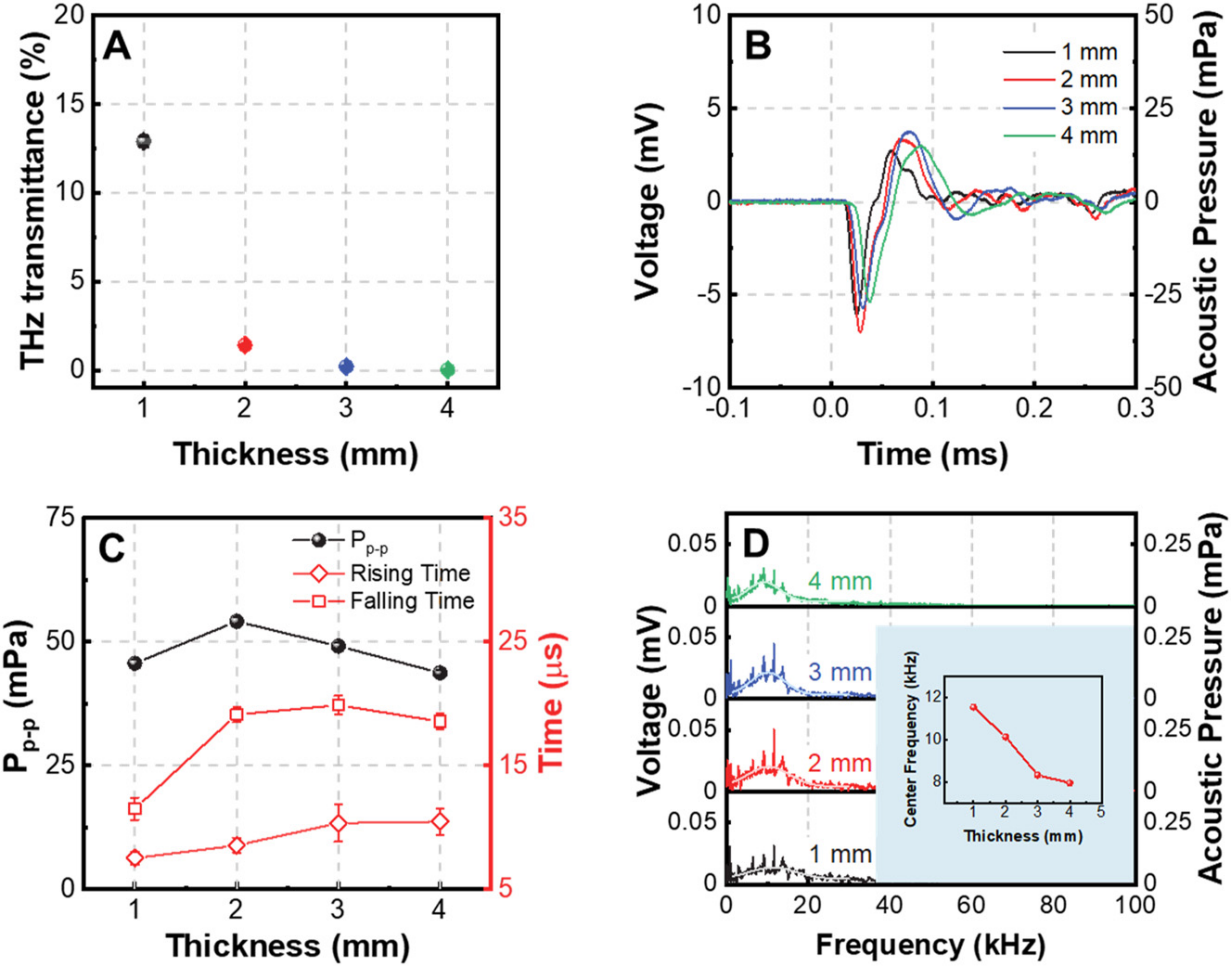
Influence of graphene foam thickness on the PTA THz detector. (A) THz transmittances of graphene foams with different thicknesses; (B) measured acoustic pulses emitted from graphene foams with thicknesses ranging from 1 to 4 mm while the THz modulation depth is kept to be 84 %; (C) dependence of the peak-to-peak acoustic pressure, rising time and falling time of the acoustic pulse on the thickness of the graphene foam; (D) acoustic frequency spectra of the acoustic pulses presented in (B).

After demonstrating the feasibility of graphene foam PTA THz detector, further investigations are performed to increase the responsivity of the detector. The thickness of the graphene foam is a critical parameter determining the responsivity of the detector because it is related with both the THz absorption and the attenuation of the acoustic pulse inside graphene foam. Therefore, the effect of the graphene foam’s thickness on the generation of the acoustic pulse is investigated. The THz transmittances of graphene foams with different thicknesses are measured using the setup in Figure S4 of the Supplementary Material [43] and the results are presented in Figure 4(A). It is found that when the thickness of the graphene foam is 2 mm or larger, the THz transmittance drops below 2 %.

The acoustic pulses emitted from the graphene foams with different thicknesses are measured when the THz modulation depth is kept as 84 %. The experimental results are presented in Figure 4(B) and the dependence of the peak-to-peak pressure of the acoustic pulse on the thickness of the graphene foam is summarized in Figure 4(C). It is seen that the optimal thickness of the graphene foam generating the most intense acoustic pulse is 2 mm. This can be explained by the fact that the graphene foam with a thickness of 2 mm can substantially absorb the THz wave and further increasing the graphene foam’s thickness may result in the additional attenuation of the acoustic wave during its propagation in the graphene foam. The dependences of the rising and falling time of the acoustic pulse on the thickness of the graphene foam are also presented in Figure 4(C). It is seen that both the rising and falling time slightly increase as the increase of the graphene foam thickness. This phenomenon can be attributed to the fact that porous material tends to have larger absorptance for higher frequency components of the acoustic wave [53–55]. As the thickness of the graphene foam increases, the high frequency components of the transmitted acoustic wave decay more severely, leading to the increase of the rising and falling time of the acoustic pulse. This can be further demonstrated by Figure 4(D) and the inset picture that the central frequency of the acoustic pulse decreases as the increase of the graphene foam thickness. The spikes in the spectra are acoustic noises generated by the interaction between the modulation laser and the silicon wafer (see the inset in Figure 3(C)). In the following, the thickness of the graphene foam is kept to be 2 mm.

**Figure 5:**
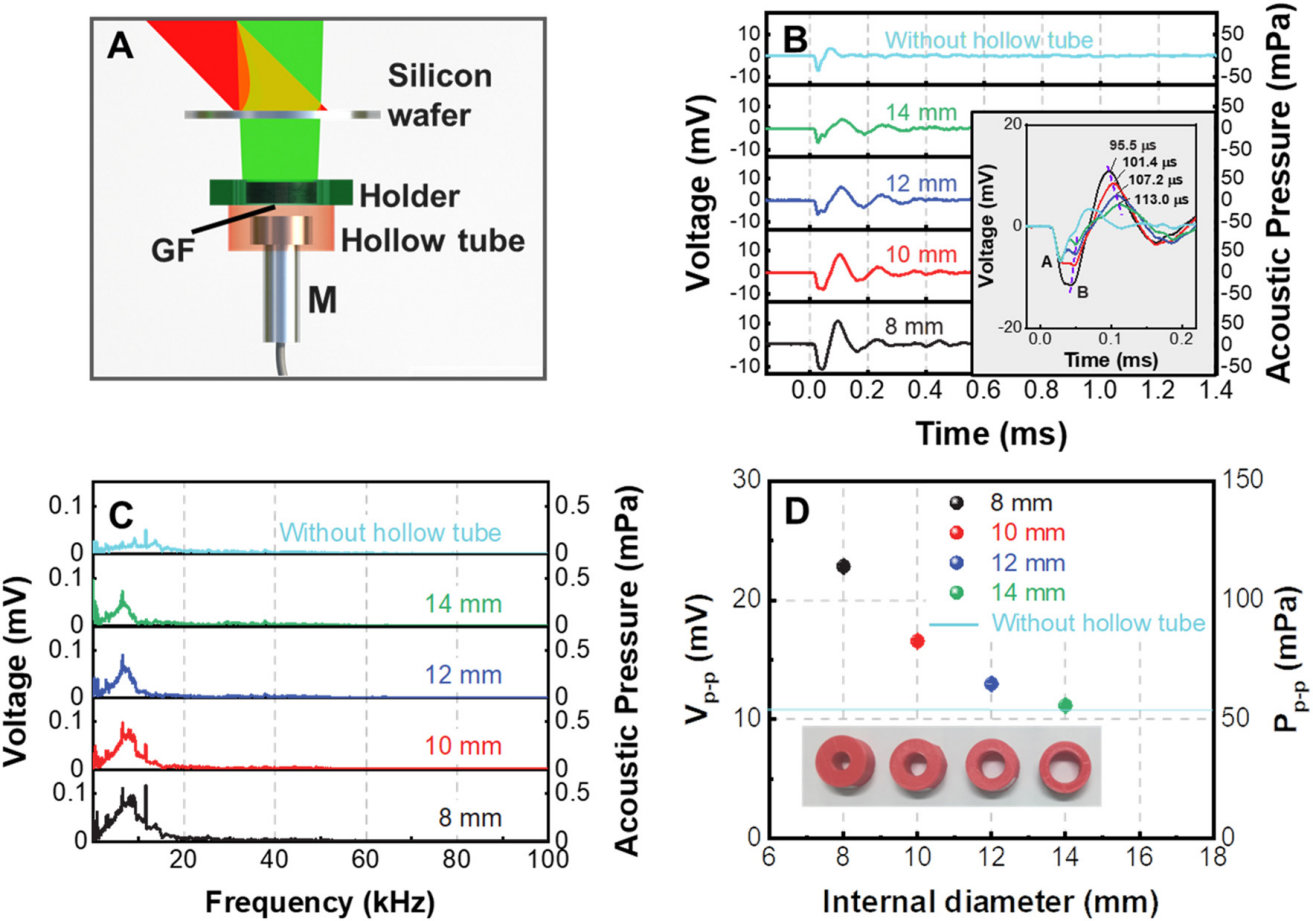
Enhancement of the responsivity of PTA THz detector using a hollow tube. (A) Configuration of the PTA THz detector using a hollow tube to increase the collection efficiency of the acoustic pulse; (B) acoustic pulse emitted from the THz irradiated graphene foam when hollow tubes with internal diameters of 8–14 mm are used; to compare the temporal profiles of these acoustic pulses, their starting points are moved to the same point as is shown in the inset; (C) spectra of the acoustic pulses in (B); (D) dependence of the peak-to-peak pressure of the acoustic pulse on the internal diameter of the hollow tube, the inset shows the photograph of the hollow tubes.

It is considered that a cylindrical hollow tube placed just behind the graphene foam may reduce the dissipation of the acoustic energy to the surroundings and increase the acoustic energy collected by the microphone. Figure 5(A) presents the configuration of the PTA THz detector equipped with a polylactide (PLA) hollow tube. Figure 5(B) shows the measured acoustic pulse emitted from the 2-mm-thick graphene foam when hollow tubes with different internal diameters are used. The THz modulation depth is kept to be 84 %. By Fourier transforming the acoustic pulses in Figure 5(B), the acoustic spectra are obtained in Figure 5(C). It is seen from Figure 5(B) that the hollow tube can prolong the time duration of the acoustic pulse and generate acoustic echo in the tail of the acoustic pulse. The hollow tube also increases the peak-to-peak pressure of the acoustic pulse which is summarized in Figure 5(D).

All these phenomena may be explained by the reflectance of the acoustic pulse on the internal wall of the hollow tube. It is noted that as the internal diameter of the hollow tube increases, the negative peak of the acoustic pulse gradually splits into two peaks, i.e. peak A and peak B in the inset of Figure 5(B). Peak A may be attributed to the acoustic pulse directly arriving at the microphone and peak B is due to the acoustic pulse reflected from the hollow tube. The positive peak time of the acoustic pulse is indicated in Figure 5(B). It is found that the differences Δ*t* between the positive peak time of the acoustic pulses are generally governed by Δ*t* = Δ*d*/*v*_s_, where Δ*d* and *v*_s_ are, respectively, the diameter difference of the hollow tubes and the sound velocity in air. This indicates that the positive peak of the acoustic pulse is mainly contributed by the reflected acoustic pulse from the internal wall of the hollow tube. The superposition of the acoustic pulses not only increases the peak-to-peak acoustic pressure, but also prolongs the duration of the acoustic pulse due to the temporal mismatch between the directly transmitting acoustic pulse and the reflected acoustic pulse. The smaller the temporal mismatch is, the larger the peak-to-peak acoustic pressure is.

In Figure 5(D) the hollow tube with an internal diameter of 8 mm can lead to the best acoustic wave enhancing effect (2.1 times) which is shown in Figure 5(D). The internal diameter of the hollow tube cannot be further decreased since the diameter of the microphone is ∼7 mm. Therefore, the temporal mismatch between the directly transmitting acoustic pulse and the reflected acoustic pulse from the wall of the hollow tube cannot be zero, which limits the further improvement of the responsivity of the detector. When the internal diameter of the hollow tube is 8 mm, the NEP of the THz detector is measured to be 533 nW/Hz^0.5^. The acoustic noise generated by the interaction between the modulation laser and the silicon wafer may be also collected by the hollow tube, causing the increase of the NEP compared with the case that no hollow tube is employed. It is also demonstrated experimentally that the wall thickness of the hollow tube has no enhancing effect on the acoustic signal which is shown in Figure S5 in the Supplementary Material [43].

**Figure 6:**
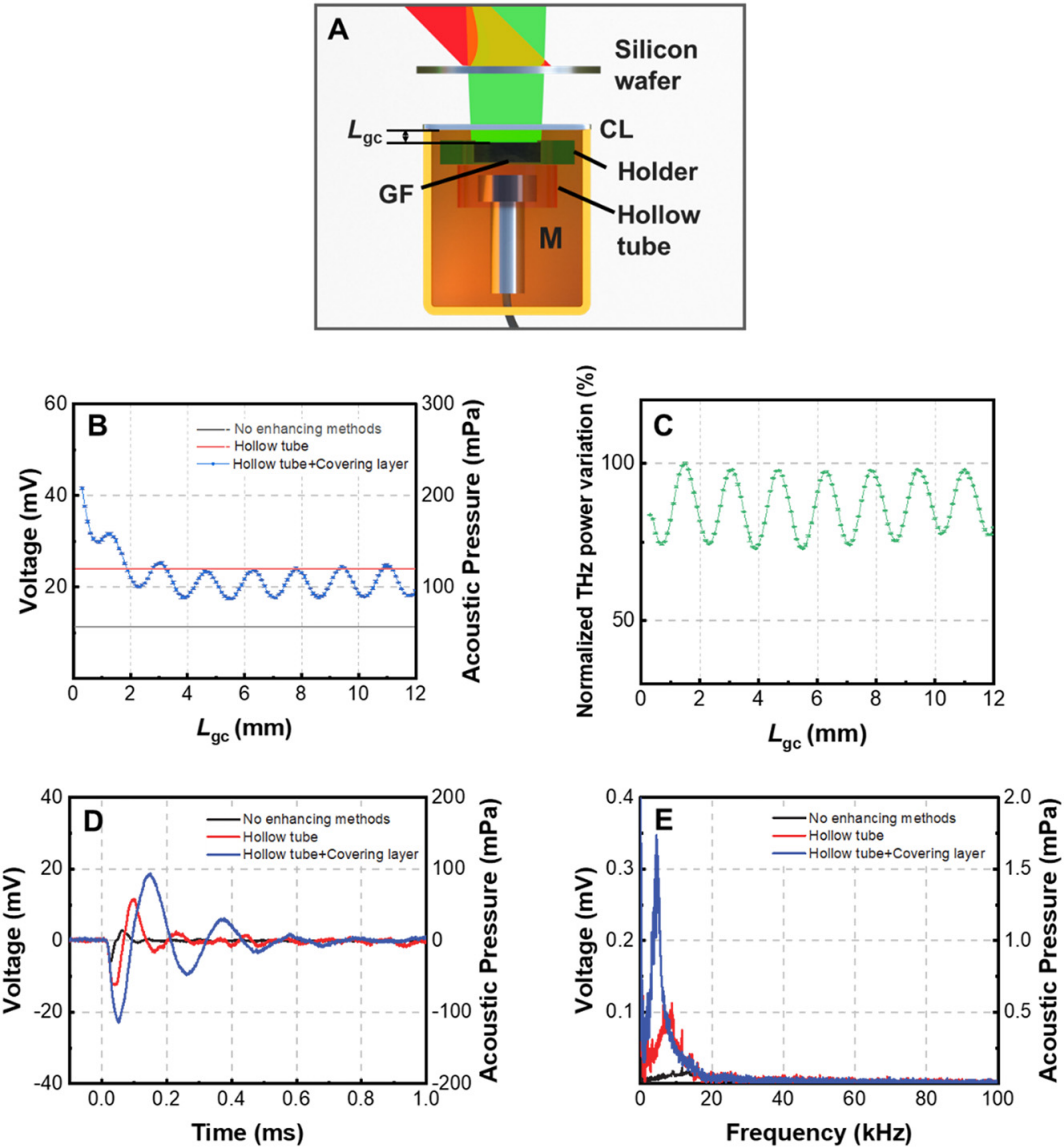
Enhancement of the responsivity of PTA THz detector by packaging. (A) Configuration of the PTA THz detector packaged inside a box; the front surface of the package box is made of the intrinsic silicon wafer with a thickness of 0.5 mm which is termed of the covering layer; (B) dependence of the acoustic pulse peak-to-peak pressure on the distance between the covering layer and the graphene foam; (C) dependence of the THz power transmitting through the graphene foam on the distance between the covering layer and the graphene foam; (D) temporal profiles of acoustic pulses emitted from graphene foam using different responsivity enhancing methods; (E) frequency spectra of acoustic pulses in (D).

In order to shield the environmental noise, the graphene foam and the microphone are packaged inside a box, which is shown in Figure 6(A). The front surface of the package box is made of the 0.5-mm-thick intrinsic silicon wafer, which is termed of the covering layer (CL). In addition to shielding noise and protecting the graphene foam from mechanical damage, it is interesting to find that the covering layer can also enhance the responsivity of the THz detector. The dependence of the peak-to-peak pressure of the acoustic pulse on the distance *L*_gc_ between the graphene foam and the covering layer (see Figure 6(A)) is measured and shown as the solid dots in Figure 6(B). The solid dot curve is measured when both the covering layer and the 8-mm-internal-diameter hollow tube are employed. It shows that as *L*_gc_ increases, the acoustic pressure oscillates periodically. The oscillation period is just the half wavelength of the THz wave. Therefore, the Fabry–Perot interferometric effect between the graphene foam and the covering layer may be responsible for the oscillation. This deduction can be demonstrated by directly measuring the THz power transmitting through the graphene foam which is shown in Figure 6(C). The THz power measured by the Schottky diode detector (WR10ZBD, Virginia Diodes Inc., equipped with a conical horn antenna (WR10CH, Virginia Diodes Inc.)) oscillates synchronously with the solid dot curve in Figure 6(B). For *L*_gc_ < 4 mm, as *L*_gc_ decreases, the acoustic pressure not only oscillates but also increases as a whole and reaches the maximum at *L*_gc_ = 0.3 mm. Smaller *L*_gc_ cannot be reached since the surface of the sample holder has contacted the covering layer at *L*_gc_ = 0.3 mm. The overall increase of the acoustic pressure may be contributed by the reflected acoustic pulse originally propagating towards the covering layer. As Figure 6(B) shows, when both the covering layer and hollow tube are used, the maximal peak-to-peak acoustic pressure is 1.7 times that only the 8-mm-internal-diameter hollow tube is employed (see red solid line in Figure 6(B)) and 3.6 times that neither the covering layer nor hollow tube is used (see black solid line in Figure 6(B)). Since the THz detector is packaged in this case, the NEP decreases to be 182 nW/Hz^0.5^.

The temporal profiles of the acoustic pulses generated by different experimental configurations are shown in Figure 6(D). The background noise for each configuration is shown in Figure S6 of the Supplementary Material [43]. The hollow tube with 8 mm internal diameter is used. When the covering layer is employed, *L*_gc_ is set to be 0.3 mm, which corresponds to the case generating the most intense acoustic pulse. It is seen that when both the covering layer and the hollow tube are employed, the rising time of the acoustic pulse is increased by 160 % compared with the case that no enhancing method is used. The increase of the rising time and time duration of the acoustic pulse is contributed by both the reflected acoustic pulse and the reflected modulated THz wave from the covering layer. Correspondingly, in Figure 6(E) it is seen that the frequency spectrum of the acoustic pulse moves to the lower frequency range due to the employment of the hollow tube and covering layer.

**Figure 7:**
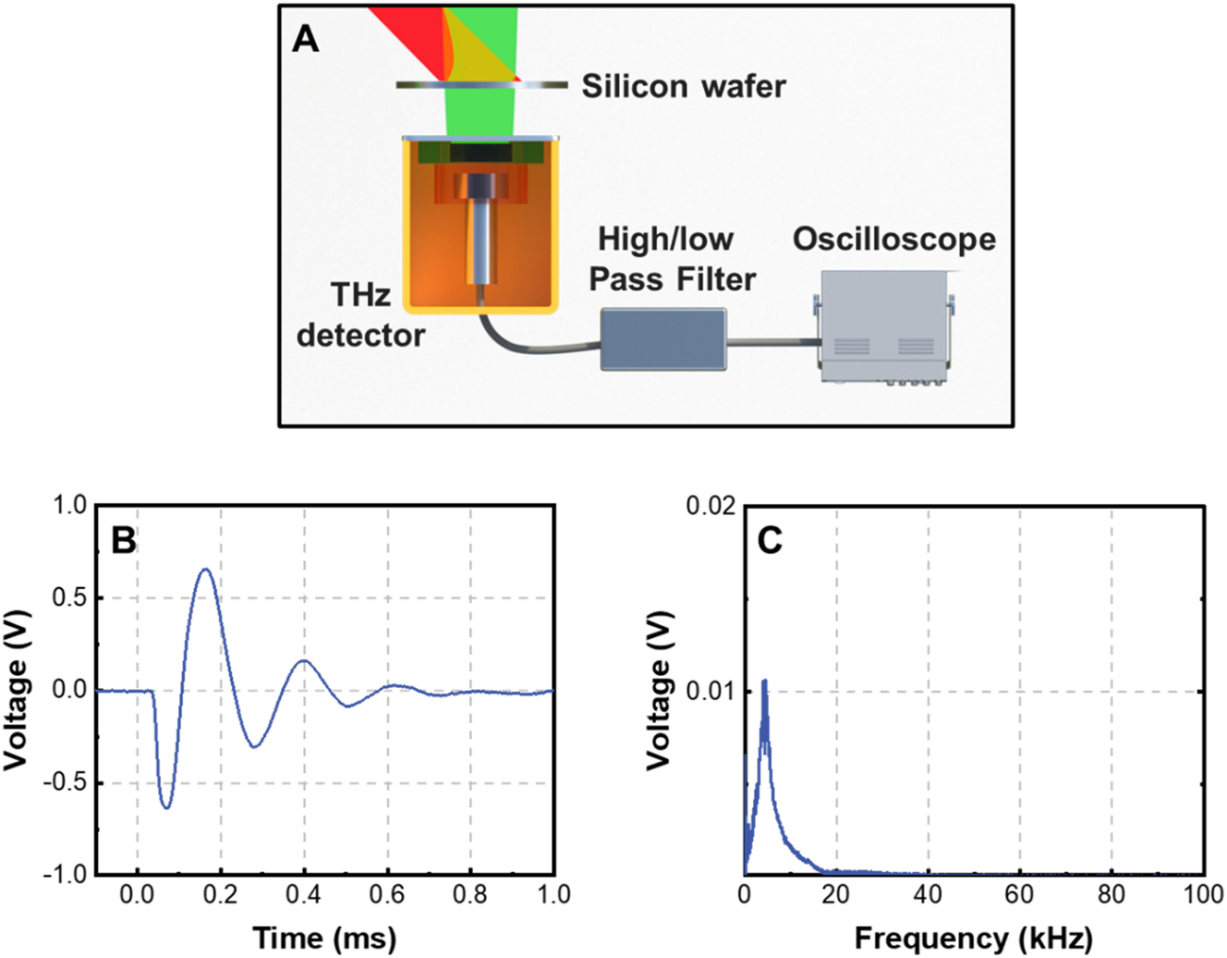
Enhancement of the responsivity of PTA THz detector using the high/low pass programmable filter. (A) Configuration of the PTA THz detector equipped with a high/low pass programmable filter; (B) temporal profile of the THz-induced acoustic pulse after electrical filtering and amplification. The internal diameter of the hollow tube is 8 mm and *L*_gc_ = 0.3 mm; the THz modulation depth is kept to be 84 %; (C) frequency spectrum of the acoustic pulse in (B).

Employing electronic filter and voltage amplifier before recording the acoustic pulse using the oscilloscope can increase the responsivity of the THz detection system, but deteriorate its NEP. The experimental setup is shown in Figure 7(A). In experiments, by using a high/low pass programmable filter (SR650, Stanford Research Systems Inc.), tens of times magnitude enhancement of the responsivity have been achieved and the temporal profile of the acoustic pulse is shown in Figure 7(B). The corresponding frequency spectrum of the acoustic pulse is presented in Figure 7(C). The responsivity of the THz detection system consisting of the THz detector and the programmable filter is 148 V/W and the NEP is determined to be 283 nW/Hz^0.5^ which is larger than the NEP of 182 nW/Hz^0.5^ for the THz detector in Figure 6(A) without using the programmable filter.

### 2.4 Broadband response of the PTA THz detector

**Figure 8:**
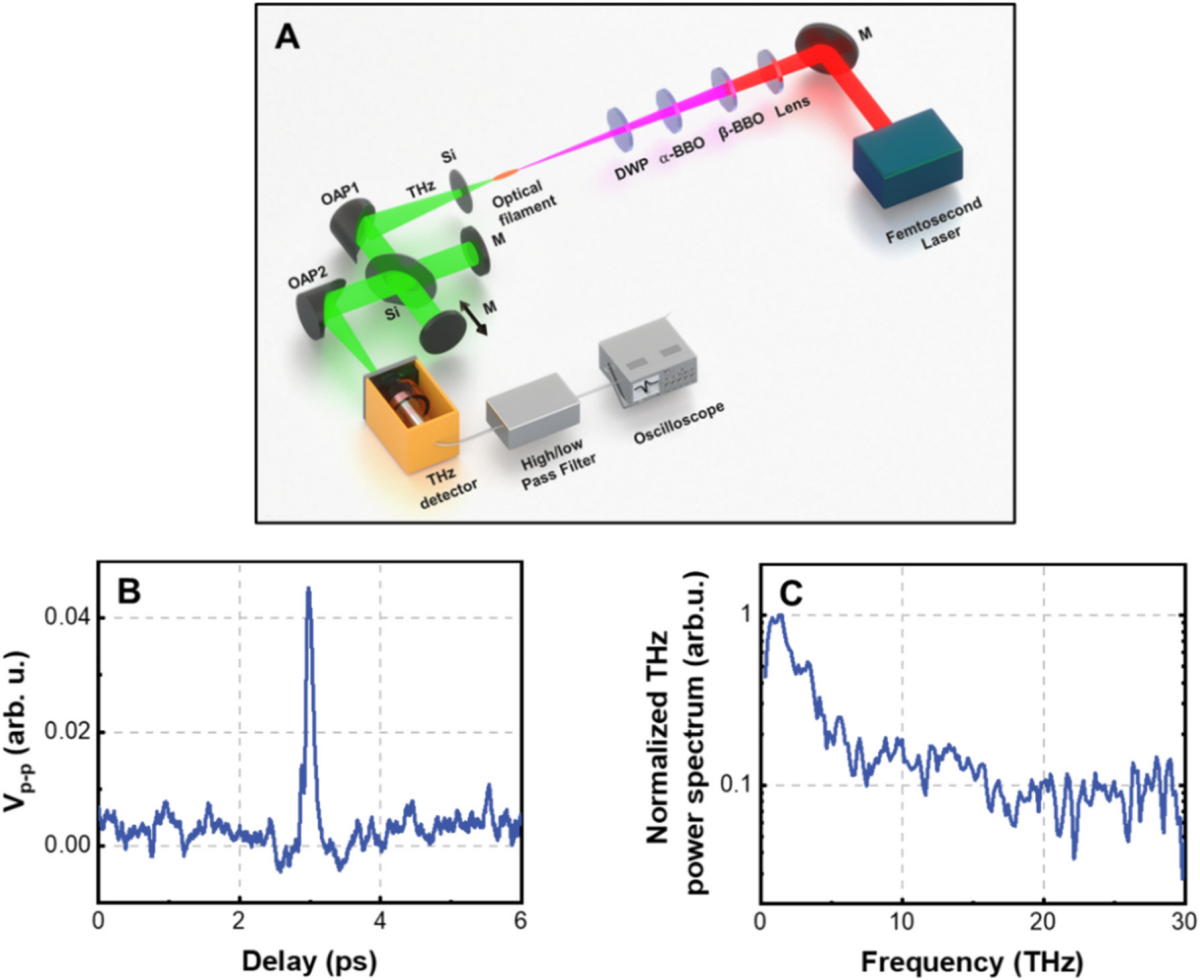
Broadband response of the PTA THz detector. (A) Experimental setup for measuring the autocorrelation of the THz pulses emitted from the dual-color femtosecond laser filament by the THz detector developed in this work; M, mirror; DWP, dual-color wave plate; Si, high resistance silicon wafer; OAP, off-axis parabolic mirror; (B) measured autocorrelation curve of THz pulses; the vertical axis is the peak-to-peak voltage measured by the microphone; (C) THz power spectrum obtained by Fourier transforming the autocorrelation curve in (B).

Finally, the PTA THz detector developed in this work is employed to measure the autocorrelation of the THz pulses generated from the dual-color (800 nm and 400 nm) femtosecond laser filament. The experimental setup is shown in Figure 8(A). 800 nm, 50 Hz, 50 fs laser pulse with single pulse energy of 3.6 mJ is employed to generate THz pulse. The focusing lens has a focal length of 300 mm. The *β*-BBO and *α*-BBO are, respectively, employed to generate the second harmonic laser beam and compensate the time delay between the dual-color beams. The dual-color wave plate is used to make the polarizations of the fundamental (800 nm) and second harmonic (400 nm) laser beams be parallel with each other. The detailed descriptions about the THz pulse generation can be found in Ref. [56]. After the optical filament, a silicon wafer is used to block the 800 nm and 400 nm laser beams and THz pulses enter into a Michelson interferometer. The autocorrelation curve of the THz pulses measured by the PTA THz detector is shown in Figure 8(B). By Fourier transforming the autocorrelation curve in Figure 8(B), the power spectrum of the THz pulse is calculated and presented in Figure 8(C). The experimental result in Figure 8(C) demonstrates that the THz detector developed in this work can be used to measure the broadband THz pulse with frequency up to tens of terahertz. In addition, our previous experimental results have demonstrated that the visible electromagnetic wave can also induce acoustic wave in graphene foam [57]. Therefore, it concludes that the graphene foam PTA detector has an ultrabroad frequency response range from 0.1 THz to visible light.

## 3 Discussion

Table 1 compares the performances of the representative commercial THz detectors and the detector studied in this work. It is found that among the THz detectors that can achieve room temperature and broadband measurement, the PTA graphene foam detector has the fastest response time (at least three orders of magnitude faster than other room temperature broadband detectors). Besides, the PTA graphene foam detector also has the second largest damage threshold. These properties make the detector studied in this work be capable of performing fast measurement in a large power range.

**Table 1:** Comparison of the representative commercial THz detectors and the detector in this work.

	Representative			Response	NEP	Damage threshold
Detector type	product	Frequency	Temperature	time (ms)	(nW/Hz^0.5^)	(mW)
Electro-optic sampling detector	EOD, Tydex [58]	0.1–4 THz	Room	–	–	20^b^
Schottky diode	QOD, VDI [59]	0.1–1 THz	Room	∼1 × 10^−7^	0.01	1
Bolometer	General purpose 4.2 K bolometer	0.15–20 THz	Low^a^	>0.6	2.5 × 10^−4^	–
	system, IR LAB [22]
Bolometer	1b, Scontel [21]	0.3–3 THz	Low^a^	∼5 × 10^−8^	5 × 10^−4^	0.05^c^
Golay cell	GC-1P/T/D, Tydex [60]	THz – visible	Room	30	0.14	0.01^d^
Pyroelectric detector	MPY-RS, WiredSense [61]	THz – visible	Room	200	0.75	15
Pyroelectric detector	THZ12D-3S-VP, Gentec [62]	0.1–30 THz	Room	3 × 10^3^	–	3.4 × 10^4^
PTA metallic film detector	TK100, Thomas Keating [40]	0.3–10 THz	Room	∼10	5 × 10^3^	500
PTA graphene foam detector	This work	THz – visible	Room	8.4 × 10^−3^	182	>3 × 10^3e^

^a^Operated at or below liquid-helium temperature. ^b^Signal-carrying beam power. ^c^Maximum power handling capacity. ^d^Recommended detected power. ^e^The damage threshold refers to the experimental results reported in Ref. [63].

Table 2 further compares the performances of the graphene-based THz detectors in literatures and the graphene foam detector in this work. It is found that compared with the recently developed graphene-based detectors, the PTA graphene foam detector can achieve good comprehensive performance (broadband, room temperature, and fast detection) using a much simpler configuration and without need of fabricating micro-electrode/antenna.

**Table 2:** Optoelectronic performances of the typical graphene-based THz detectors.

				Response time		Micro-electrode/
Material	Mechanism	Frequency	Temperature	(ms)	NEP (nW/Hz^0.5^)	antenna	Ref.
Graphene	Photo-conduction	∼0.1 THz	Room	0.02	0.5	Required	[64]
Graphene	Schottky junction	∼0.1 THz	Room	1	–	Required	[65]
Graphene	Bolometer	1.36–37.5 THz	Room	5 × 10^−8^	–	Required	[32]
Graphene	FET	∼0.3 THz	Room	<5 × 10^−3^	0.163	Required	[35]
Graphene	FET	∼0.95 THz	Room	∼1 × 10^−8^	166	Required	[66]
Graphene	FET	0.001–1.1 THz	Room	–	0.03	Required	[67]
Graphene	FET	THz – visible	Room	2.7 × 10^−4^	0.048	Required	[31]
Graphene	PTE	0.1−10 THz	Room	<1 × 10^−6^	≤0.12	Required	[68]
Graphene	PTE	∼2.52 THz	Room	1.1 × 10^−7^	1.1	Required	[34]
Graphene	PTE	THz – visible	Room	∼1 × 10^3^	–	Not required	[69]
Graphene foam	PTE	THz – visible	Room	23	7 × 10^3^	Not required	[37]
Graphene foam	PTA	THz – visible	Room	8.4 × 10^−3^	182	Not required	This work

The graphene foam THz detector developed in this paper could simultaneously achieve room temperature, full bandwidth and fast THz detection, which is requisite in THz biomedical diagnoses, such as the skin lesions and tumor margin assessment by THz pulse imaging (TPI) [70] or THz coherent tomography (TCT) [71], and the investigation of protein folding dynamics by high speed THz spectroscopy [72].

In conclusion, a novel THz detection method based on the PTA effect of graphene foam is proposed in this paper in which the THz wave is measured by a microphone with audible frequency response. This THz detection method not only has the advantages of the photo-thermal THz detector, such as room temperature and full bandwidth, but also possesses a fast response time in microsecond time scale. Besides, no antenna and electrode are required to fabricate which simplifies the configuration and decreases the cost of the detector. It is believed that the microphone used to detect the THz wave can be replaced by the fiber optic acoustic sensor [42, 73], which makes the fabrication of an ultra-low-cost, fast-response, THz array detector become possible. The detection unit may be constructed simply by a piece of graphene foam and an optical fiber. The newly developed PTA THz detection scheme has good comprehensive performances among both the commercial THz detectors and recently developed detectors in laboratory.

## 4 Materials and methods

In this work, the graphene foam is prepared by the solvothermal method using graphene oxide as raw material. The detailed synthetic procedures can be found elsewhere [36]. According to the Raman results in Figure 1(C), it is seen that limited by the preparation method, the quality of the graphene foam is medium. However, according to the results in the literature, further increasing the quality of graphene foam, i.e. reducing the defects and the graphene layer, represented by the reduction of D peak and the enhancement of 2D peak, may increase the effective dielectric constant of graphene foam and increase the THz reflectance on the graphene foam, which is adverse to the THz wave absorption [63].

## Supplementary Material

Supplementary Material Details
