# Supplementary material for: Detecting terahertz wave by microphone based on the photoacoustic effect in graphene foam
Source: Nanophotonics. 2023 Jun 14;12(15):3053–67. doi: 10.1515/nanoph-2023-0026 (PMC11502112; doi:10.1515/nanoph-2023-0026)
Supplement: Supplementary file 1 — Supplementary Material Details [file j_nanoph-2023-0026_suppl_001.pdf]

Supplementary Materials for

Detecting terahertz wave by microphone based on the photoacoustic effect in graphene foam

Nan Zhang [1†], Tingyuan Wang [1†], Guanghao Li [2], Lanjun Guo [1], Weiwei Liu [1*], Ziyuan Wang [3], Guanghui Li [3], Yongsheng Chen [3*]

[1] Institute of Modern Optics, Nankai University, Tianjin Key Laboratory of Micro-scale Optical Information Science and Technology, Tianjin 300350, China.

[2] National Institute for Advanced Materials, Tianjin Key Laboratory of Metal and Molecule Based Material Chemistry, School of Materials Science and Engineering, Nankai University, Tianjin 300350, China.

[3] Key Laboratory for Functional Polymer Materials and The Centre for Nanoscale Science and Technology, Institute of Polymer Chemistry, Synergetic Innovation Center of Chemical Science and Engineering (Tianjin), College of Chemistry, Nankai University, Tianjin 300071, China.

†These authors contribute equally to this work.

*liuweiwei@nankai.edu.cn

*yschen99@nankai.edu.cn

1 Detection speed of the PTA graphene foam detector

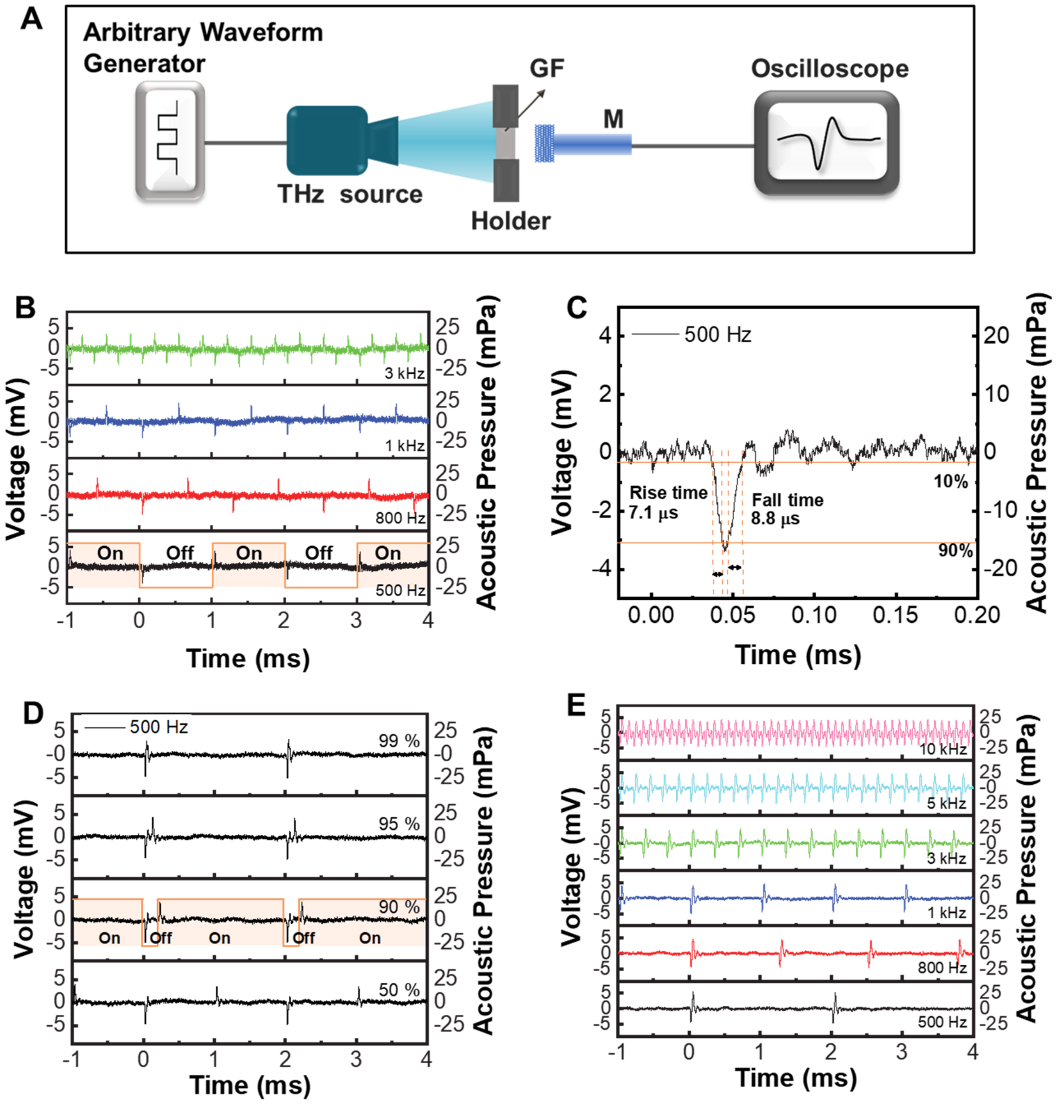

Fig. S1. Detection speed of the PTA graphene foam detector. (A) Experimental setup for testing the detection speed of the PTA THz detector; (B) acoustic pulses emitted from the graphene foam under the irradiation of the modulated THz waves with duty cycle of 50% and various modulation frequencies; (C) temporal profile of the acoustic pulse generated by the off edge of the modulated THz wave; (D) effect of the duty cycle of the modulated THz wave on the acoustic pulse emitted from the graphene foam; (E) acoustic pulses emitted from the graphene foam under the irradiation of the modulated THz waves with modulation frequencies from 500 Hz – 10 kHz.

The experimental setup is shown in Fig. S1(A). An arbitrary waveform generator (DG3101A, Rigol Inc.) is employed to generate the periodic square wave with a duty cycle of 50%. Under the modulation of the square wave, periodically modulated THz wave synchronizing with the square wave is emitted from the THz source (0.1 THz, 86 mW, Terasense Inc.). The modulated THz wave irradiates the graphene foam and the induced acoustic pulses are detected by the microphone (378C01, PCB Inc.). Fig. S1(B) presents the acoustic pulse trains emitted

from the graphene foam under the irradiation of THz waves with various modulation frequencies. Taking the case with 500 Hz modulation frequency as an example, both the off and on edges of THz wave (see the orange line in Fig. S1(B)) can induce acoustic pulses in graphene foam. The off and on edges of the THz wave respectively generate acoustic pulses with negative and positive acoustic pressures. The acoustic pulse generated by the off edge of the 500 Hz modulated THz wave is shown in Fig. S1(C). It is seen that the sum of the rising and falling time of the acoustic pulse is shorter than 16 μs.

The experimental results in Fig. S1(D) show that when the duty cycle of the modulated THz wave increases to 99%, the two acoustic pulses respectively generated by the off and on edges of the THz wave merge to one acoustic pulse whose duration is no longer than 40 μs. Fig. S1(E) presents the acoustic pulse train emitted from the graphene foam under the irradiation of the modulated THz waves with modulation frequencies of 500 Hz – 10 kHz. In Fig. S1(E), the off duration in each period of the modulation square wave generated by the arbitrary waveform generator is kept to be 20 μs. The 10 kHz acoustic pulses are clearly resolved in Fig. S1(E). Therefore, the PTA THz detector in this work can detect a THz pulse train with repetition rate ⩾10 kHz.

Using the data shown in Fig. S1(E), the standard deviation σ of the acoustic pulse's peak-to-peak value is summarized in Table S1. From Table S1, it concludes that the modulation frequency does not affect the accuracy of the detector when the modulation frequency is no more than 10 kHz.

Table S1 Dependences of the standard deviation σ of the acoustic pulse's peak-to-peak value on the modulation frequency. The data are obtained from Fig. S1(E).

Modulation frequency (kHz)	σ (mV)
0.5	0.22
0.8	0.19
1	0.22
3	0.20
5	0.23
10	0.25

2 Cross-sectional intensity profile of the THz wave

The THz wave's intensity distribution is measured using the setup in Fig. S2(A). A zero-biased Schottky diode detector (WR10ZBD, Virginia Diodes Inc.) without the conical horn antenna is employed to measure the cross-sectional intensity profile of the THz wave on the silicon wafer. The size of the detection area of the Schottky diode detector is 2.54 × 1.27 mm^2, which is represented by the orange rectangle in Fig. S2(A). To avoid damaging the Schottky diode detector, a 0.5 mm-thick doped silicon wafer with a resistivity of 2.3 W · cm and a diameter of 15 cm is inserted between the two off-axis parabolic mirrors (OAP). The included angle between the normal direction of the wafer and the propagation direction of THz wave is 45° which can prevent the reflected THz wave from returning to the THz source. The THz transmittance of the silicon wafer is 9%. The normalized cross-sectional intensity profile of the THz wave on the silicon wafer is measured via the point-by-point scanning manner and the result is shown in Fig. S2(B).

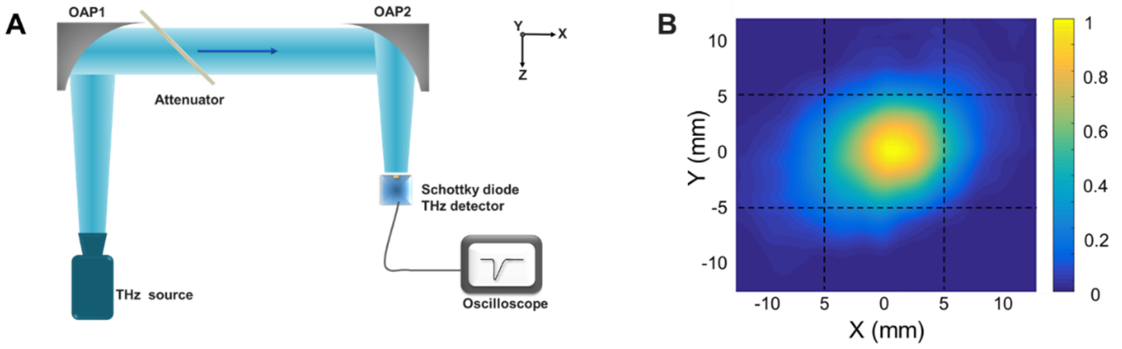

Fig. S2. THz wave's intensity distribution. (A) Experimental setup for measuring the cross-sectional intensity profile of the THz wave; OAP: off-axis parabolic mirror. (B) normalized cross-sectional intensity profile of THz wave on the silicon wafer.

3 Cross-sectional intensity profile of the modulation laser

The modulation laser's intensity distribution is measured using the setup in Fig. S3(A). Three optical wedges are used to steer the laser beam and attenuate the laser power such that the laser beam cannot saturate the CCD camera (M2S132M-H2, DO3THINK Inc.). A lens with a focal length of 300 mm is used to shrink the laser spot, which guarantees the laser spot can be collected completely by the CCD camera.

The measurement result is shown in Fig. S3(B). It should be noted that in Fig. S3(B) the lens' shrinkage effect on the beam spot has been considered and the scale bar indicates the actual size of the beam spot. The incident angle of the modulation laser on the silicon wafer is measured to be 45°. Therefore, by projecting the cross-sectional profile in Fig. S3(B) on the silicon wafer, the intensity profile of the modulation laser on the silicon wafer can be obtained and shown in Fig. S3(C).

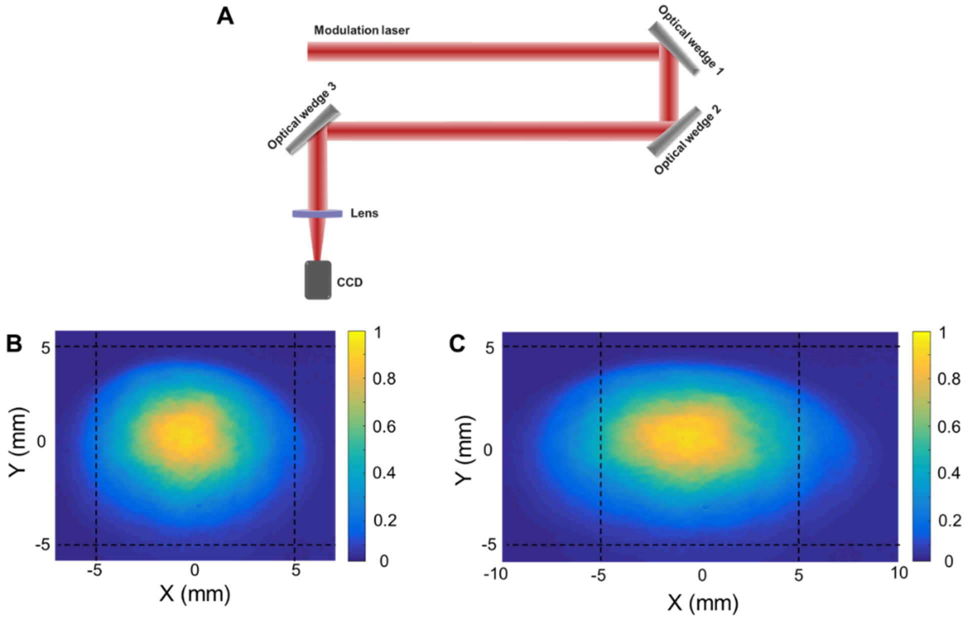

Fig. S3. Intensity distribution of modulation laser. (A) Experimental setup for measuring the cross-sectional intensity distribution of the modulation laser; (B) normalized beam intensity distribution; (C) normalized beam intensity distribution on the silicon wafer.

4 Measurement method of the THz transmittance of graphene foam

The experimental setup for measuring the THz transmittance of graphene foam is presented in Fig. S4. The Schottky diode detector (WR10ZBD, Virginia Diodes Inc.) is employed to measure the THz power respectively with and without the graphene foam in the sample holder to determine the THz transmittance of the graphene foam. In order to receive all the THz power transmitting through the graphene foam, a conical horn antenna with aperture diameter of 16.3 mm (WR10CH, Virginia Diodes Inc.) is added in front of the detector. It should be noted that the THz wave cannot transmit through the sample holder. The THz transmittances of the graphene foams with thicknesses from 1 mm to 4 mm are obtained and shown in Fig. 4(A) of the main text of this paper.

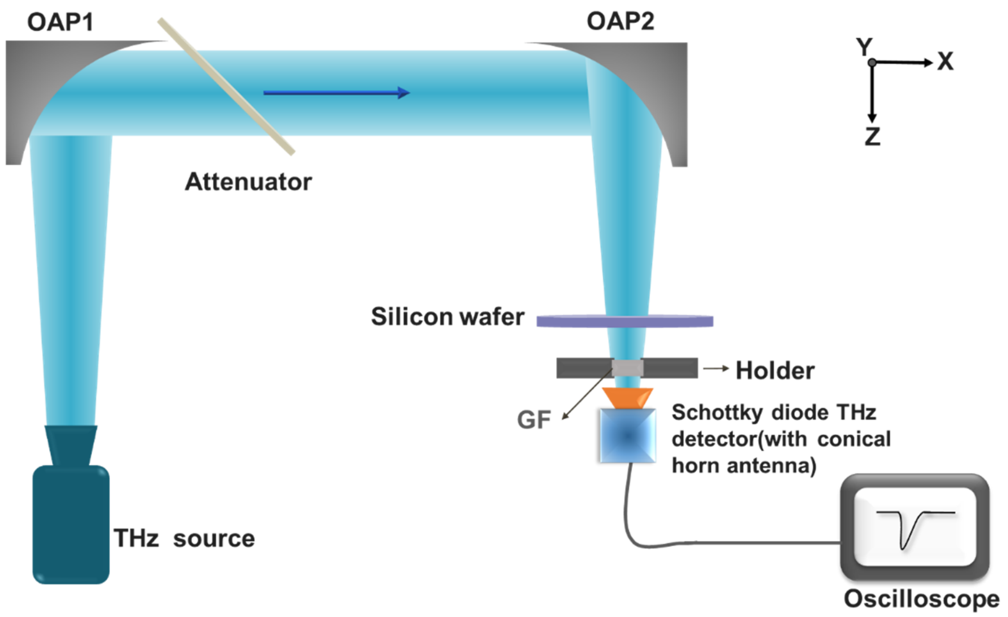

Fig. S4. Experimental setup for measuring the THz transmittance of graphene foam.

5 Effect of the wall thickness of the hollow tube on the detector's responsivity

Three polylactide (PLA) hollow tubes with different wall thicknesses are employed to investigate the wall thickness' effect on the THz wave induced acoustic pulse. The PLA tubes respectively have wall thickness of 4 mm, 5 mm and 6 mm, while the internal diameters of these tubes are kept to be 10 mm. The pictures of the tubes can be seen in the inset of Fig. S5(A). During the experiments, the modulation laser with an average power of 30 mW is employed, leading to a ~84% modulation depth of the THz wave. Therefore, the variation of the THz power is calculated to be 8.7 mW. The dependence of the peak-to-peak voltage, i.e. the peak-to-peak pressure of the THz wave induced acoustic pulse measured by the microphone on the wall thickness is shown in Fig. S5(A). The temporal profiles of the acoustic pulses and their frequency spectra are shown in Figs. S5(B) and S5(C).

Each data point in Fig. S5(A) is obtained by repeatedly measuring the acoustic pulses for 20 times. Also, each curve in Fig. S5(B) is obtained by averaging the measured 20 acoustic pulses. The frequency spectra in Fig. S5(C) are calculated by Fourier transforming the temporal profile of the acoustic pulse in Fig. S5(B). It concludes that the wall thickness of the hollow tube has negligible effect on the amplitude and frequency spectrum of the THz wave induced acoustic pulse.

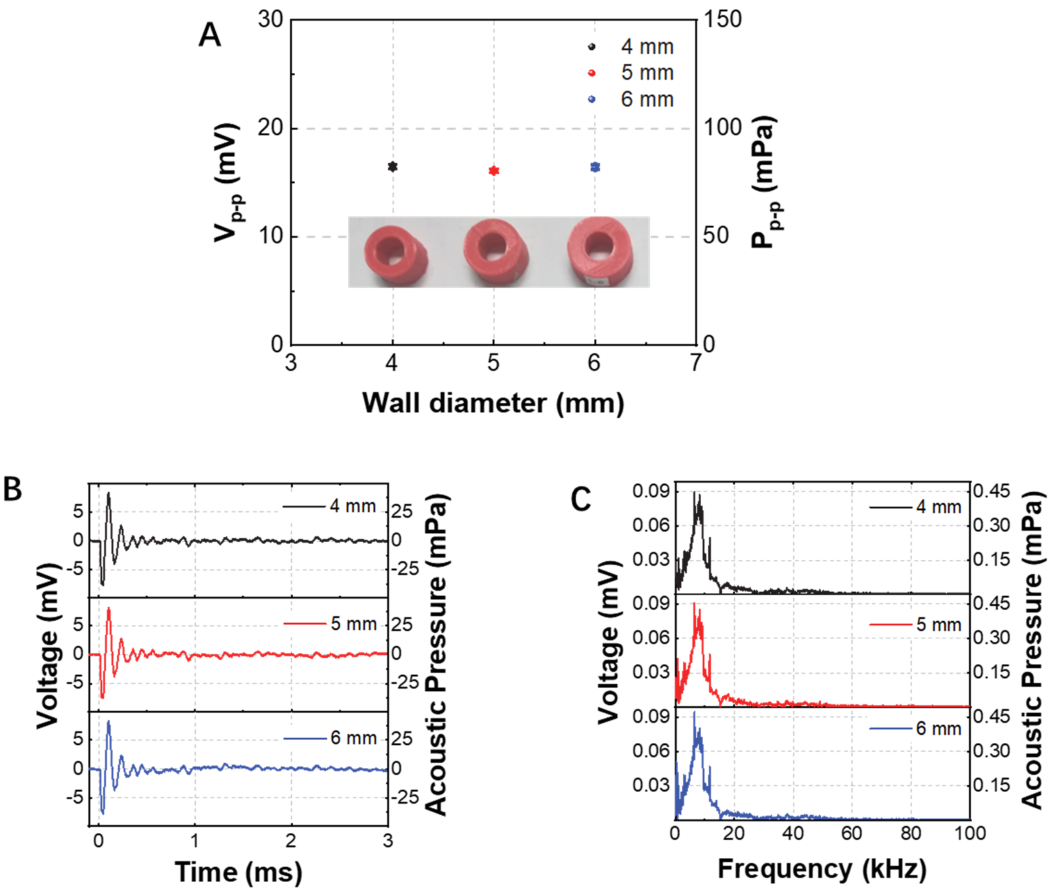

Fig. S5. Influence of the wall thickness of the hollow tube on the PTA graphene foam detector. (A) Dependence of the peak-to-peak pressure of the THz wave induced acoustic pulse on the wall thickness of the hollow tube; (B) temporal profiles of the acoustic pulses emitted from the graphene foam when hollow tubes with wall thicknesses of 4 mm, 5 mm and 6 mm are respectively used; (C) corresponding frequency spectra of the acoustic pulses in (B).

6 Acoustic pulses emitted from graphene foam under different apparatus configurations

Fig. S6 presents the acoustic pulses detected by the microphone when different responsivity enhancement methods are employed. It is seen that to excite acoustic pulses, the modulation laser and the THz wave must be simultaneously employed, i.e. the modulated THz wave is requisite for generating acoustic pulses. It is also noted that the acoustic noise generated by the interaction between the modulation laser and the silicon wafer (see the red curves in Fig. S6) can be completely blocked by the packaging box. Thus, it can be deduced that the packaging box effectively shields the external acoustic noise.

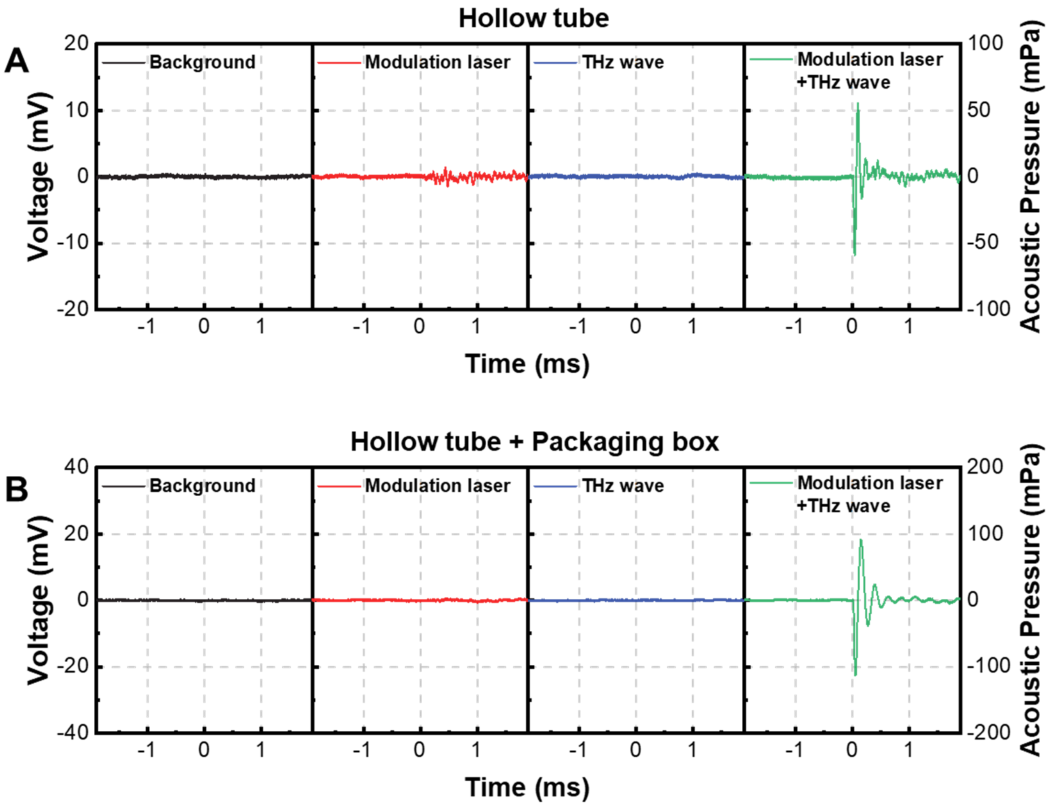

Fig. S6. Acoustic signal measured by the microphone under different experimental conditions. (A) The hollow tube is employed; (B) both the hollow tube and packaging box are employed. The power of the modulation laser is kept to be 30 mW and the THz modulation depth is 84%. The four different experimental conditions are as follows: 1) neither the modulation laser nor THz wave is employed, 2) only the modulation laser is employed, 3) only the THz wave is employed, 4) both the modulation laser and THz wave are employed.
